# Tuning Selectivity of Fluorescent Carbon Nanotube-Based Neurotransmitter Sensors

**DOI:** 10.3390/s17071521

**Published:** 2017-06-28

**Authors:** Florian A. Mann, Niklas Herrmann, Daniel Meyer, Sebastian Kruss

**Affiliations:** 1 Institute of Physical Chemistry, Göttingen University, 37077 Göttingen, Germany; florian.mann@med.uni-goettingen.de (F.A.M.); niklas.herrmann1@stud.uni-goettingen.de (N.H.); daniel.meyer@uni-goettingen.de (D.M.); 2 Center for Nanoscale Microscopy and Molecular Physiology of the Brain (CNMPB), 37073 Göttingen, Germany

**Keywords:** carbon nanotube, biosensor, fluorescence, DNA, neurotransmitter, affinity

## Abstract

Detection of neurotransmitters is an analytical challenge and essential to understand neuronal networks in the brain and associated diseases. However, most methods do not provide sufficient spatial, temporal, or chemical resolution. Near-infrared (NIR) fluorescent single-walled carbon nanotubes (SWCNTs) have been used as building blocks for sensors/probes that detect catecholamine neurotransmitters, including dopamine. This approach provides a high spatial and temporal resolution, but it is not understood if these sensors are able to distinguish dopamine from similar catecholamine neurotransmitters, such as epinephrine or norepinephrine. In this work, the organic phase (DNA sequence) around SWCNTs was varied to create sensors with different selectivity and sensitivity for catecholamine neurotransmitters. Most DNA-functionalized SWCNTs responded to catecholamine neurotransmitters, but both dissociation constants (*K*_d_) and limits of detection were highly dependent on functionalization (sequence). *K*_d_ values span a range of 2.3 nM (SWCNT-(GC)_15_ + norepinephrine) to 9.4 μM (SWCNT-(AT)_15_ + dopamine) and limits of detection are mostly in the single-digit nM regime. Additionally, sensors of different SWCNT chirality show different fluorescence increases. Moreover, certain sensors (e.g., SWCNT-(GT)_10_) distinguish between different catecholamines, such as dopamine and norepinephrine at low concentrations (50 nM). These results show that SWCNTs functionalized with certain DNA sequences are able to discriminate between catecholamine neurotransmitters or to detect them in the presence of interfering substances of similar structure. Such sensors will be useful to measure and study neurotransmitter signaling in complex biological settings.

## 1. Introduction

Neurotransmitters are essential for basic functions of the human body and especially for chemical signaling in neuronal circuits of the brain. However, their mode of action is widely unexplored due to a lack of tools to measure their concentration profiles in a spatiotemporal manner. In the past decades several analytical methods have been developed to measure neurotransmitter concentrations [1,2,3,4,5]. Those methods range from magnetic resonance imaging with contrast agents for neurotransmitters to electrochemical approaches, but most of them lack either high spatial or temporal resolution or they are not compatible with biological environments [2,3,4,6]. The detection of neurotransmitters is very challenging because of several limiting conditions. First, many neurotransmitters are small molecules that share structural homologies with each other and with additional interfering substances in the brain or in the cell culture. Second, during exocytotic events only 100–100,000 molecules are released within milliseconds [7]. Finally, the most prominent release sites, i.e., synapses, are very small (300 × 300 × 20 nm^3^) and not easily accessible by macroscopic probes [8]. All these hallmarks of neural networks indicate that neurotransmitter sensors should be sensitive/selective (single-molecule level), label-free, small (nanoscale), fast (milliseconds), and non-invasive (e.g., optical).

Classical approaches to detect (redox-active) neurotransmitters are electrochemical methods such as amperometry or cyclic voltammetry [9,10]. Many biological studies were only possible because these methods provided quantitative information about neurotransmitter concentrations around cells, in brain slices and in vivo [2,11]. However, electrochemical methods are limited to molecules that can be oxidized at the electrode (e.g., dopamine or serotonin) or make use of enzymatic reactions [12]. Therefore, important neurotransmitters, such as glutamate or γ-aminobutyric acid (GABA), cannot be detected. Moreover, electrodes are large compared to the site of neurotransmitter release, which limits simultaneous and parallel/spatial detection. Another approach is based on modifying biological recognition units of neurotransmitters and conjugating them with fluorescent dyes. This method was used to engineer GABA sensors and glutamate sensors [13,14]. Recently, glutamate was detected by using green-fluorescent protein (GFP)-conjugated glutamate receptors that can be also transfected into cells [15]. The disadvantage of this approach is the need for cell transfection and manipulation, which is difficult in complex primary biological samples and in vivo. Additionally, sensing is restricted to the cell surface and, therefore, diffusion characteristics cannot be assessed.

Nanomaterials are promising building blocks for neurotransmitter sensors/probes [1]. Among the different materials carbon nanotubes attract a lot of interest due to their unique optoelectronic properties. Semiconducting single-walled carbon nanotubes have a bandgap that leads to near infrared (NIR) fluorescence [16]. SWCNTs can be non-covalently decorated with an organic phase generating the desired specificity for the molecular target. Examples for sensors of this class range from small molecule analytes, like neurotransmitters, sugars, and explosives, to miRNA or proteins [17,18,19,20,21,22,23]. These sensors combine specificity for the target generated by the organic corona-phase and the advantageous properties of SWCNTs for optical sensing and imaging [16,24,25,26]. These advantages are, among others, the extraordinarily high photostability compared to organic fluorophores, the absence of fluorescence blinking, as well as the large Stokes-shift of >400 nm allowing for low-background imaging in the biologically-transparent near-infrared (NIR) window [16]. Such reversible sensors have been used to detect the efflux of dopamine from cells by imaging many of them at the same time [5]. This approach enabled a spatial resolution that was not possible before with electrochemical approaches.

The organic phase (corona) around these sensors plays a central for molecular recognition and signal transduction. The mechanism and the reasons for selectivity are, however, still not completely understood. Fluorescence changes were attributed to conformational changes, redox chemistry and free surface area [5,27,28]. Another possible mechanism could be a change of exciton diffusion upon binding of an analyte [29].

Although SWCNT-based sensors have shown great potential for interesting applications, there are still several obstacles to overcome with respect to specificity, affinity, and kinetics. Especially kinetics (rate constants) and dissociation constants play decisive roles in the fast detection of neurotransmitters [30]. These issues have not yet been taken into account so far, but will be important to enable applications in chemically-complex environments.

**Figure 1:**
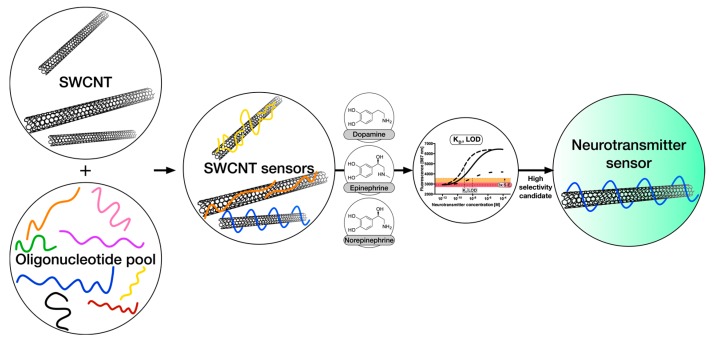
Strategy to measure and optimize selectivity and sensitivity of neurotransmitter sensors. Candidate sensors are synthesized from single-walled carbon nanotubes (SWCNTs) and DNA oligonucleotides and their responses to the neurotransmitters dopamine, epinephrine and norepinephrine are quantified. Crucial for the success of these sensors is the discrimination between different, but chemically very similar, neurotransmitters. In this work, a set of different DNA-oligonucleotides is tested as an organic phase and corresponding sensor properties, such as *K*_d_-values, are evaluated to find the most selective and robust sensors.

In this work, we address remaining challenges of neurotransmitter detection with carbon nanotube-based sensors by varying the organic phase around the SWCNT scaffold and evaluating the resulting dissociation constants and limits of detection for relevant neurotransmitters. This approach (Figure 1) enables us to identify organic phases (DNA sequences) that impart the best sensor performance (selectivity, sensitivity at relevant concentrations, dynamic range, etc.).

## 2. Materials and Methods

Materials: Unless stated otherwise, all chemicals and oligonucleotides were purchased from Sigma Aldrich (Taufkirchen, Germany) or TCI (Eschborn, Germany). For epinephrine and norepinephrine, the racemic mixture was used.

Dispersion and functionalization of carbon nanotubes: To generate a stable single-walled carbon nanotube (SWCNT) dispersion under physiological conditions, 0.5 mg oligonucleotide was added to 0.5 mg of 6,5-chirality enriched SWCNT (Sigma Aldrich, Product No.: 773735) in 0.5 mL 1× phosphate-buffered saline (PBS). The resulting suspension was first tip sonicated for 10 min (Fisher Scientific™ Model 120 Sonic Dismembrator, 20% amplitude) and subsequently centrifuged at 16,100 g (2 × 30 min) to remove large bundles, aggregates or metal catalysts remaining from SWCNT synthesis. The supernatant containing individualized SWCNTs was used as a stock solution for downstream experiments after absorbance measurements and estimation of nanotube concentration using the molar extinction coefficient at 991 nm [31]. The solutions corresponding to the different DNA-sequences were adjusted in concentration according to the collected absorbance spectra.

Spectroscopy of SWCNT-DNA complexes: NIR absorbance spectra were measured with a UV-VIS-NIR spectrometer (JASCO V-670, Spectra Manager Software) using a 10 mm-path cuvette.

NIR fluorescence spectra were recorded on a Shamrock 193i spectrograph (Andor Technology Ltd., Belfast, Northern Ireland) coupled to an Olympus BX53 microscope using an exposure time of 10 s and a slit width of 500 μm and an Andor iDus InGaAs 491 array NIR detector. SWCNTs were excited at 560 nm.

NIR-fluorescence dose-response curves: Ten microliters of the corresponding and freshly-prepared catecholamine ·HCl solution (0, 100 pM, 1 nM, 10 nM, 100 nM, 1 μM, 10 μM, 100 μM) in 1× phosphate-buffered saline (PBS) were added to 90 μL of DNA-suspended SWCNTs (0.1 nM, in 1× PBS) in a 96-well plate format. The fluorescence counts were averaged from triplicates and plotted against the neurotransmitter concentration on a log-scale.

Extraction of dissociation constants from dose-response curves: The NIR-fluorescence dose-response curves were fitted using Equation (2). From this fit the dissociation constant and its 95% confidence interval were extracted for each combination of SWCNT-DNA sensor and catecholamine. In the case of dopamine, 100 μM values were neglected for *K*_d_ estimation, as dopamine is prone to oxidation and polymerization at higher concentrations [32,33], which was previously shown to have an effect on SWCNT-DNA fluorescence modulation [17].

Near infrared microscopy of immobilized SWCNT-DNA sensors: Twenty microliters of the 0.1 nM SWCNT-DNA (SWCNT-(GT)_10_, SWCNT-(GA)_15_) solution were incubated on (3-aminopropyl)triethoxysilane (APTES)-pre-activated glass bottom Petri dishes (1 wt % APTES/H_2_O in EtOH, 1 h) overnight at 4 °C. After a 1× PBS wash, NIR imaging was carried out on an Olympus BX53 microscope (Olympus K.K., Tokyo, Japan) using a 60× oil-immersion objective and a Xenics^®^ Xeva-1.7-320 NIR camera (Xenics, Heverlee, Belgium). The frame-rate was set to 0.5 fps. After approx. 10 frames, 20 μL 100 nM norepinephrine solution was added to the adsorbed SWCNT-DNA. Dopamine was added after 50 additional frames in the same volume and concentration to yield 40 μL containing 50 nM of each neurotransmitter.

Dose-response measurements of immobilized SWCNT-DNA sensors: One-hundred microliters of a 1.1 μM SWCNT-(GT)_10_ solution in 1× PBS was incubated on glass-bottom Petri dishes for one hour at 4 °C. After a 1× PBS wash, NIR imaging was carried out on an Olympus BX53 microscope using a 60× oil-immersion objective and a Xenics^®^ Xeva-1.7-320 NIR camera. The frame-rate was set to 0.5 fps. Approx. every 30 frames, 5 μL of increasing dopamine concentrations (a = 1 nM, b = 10 nM, c = 100 nM, d = 1 μM, e = 10 μM) were added to a 50 nM norepinephrine solution (in 1× PBS, 500 μL) on top of the adsorbed SWCNT-DNA.

## 3. Results

### 3.1. Preparation and Optical Properties

**Figure 2:**
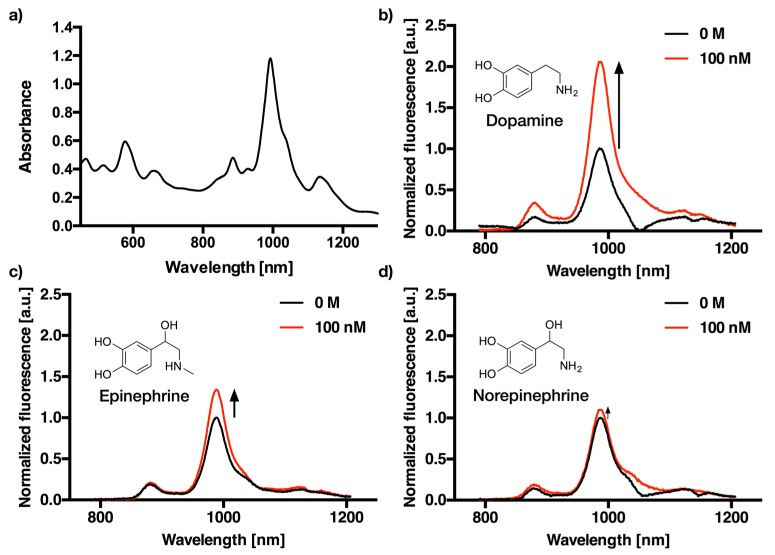
Absorbance and fluorescence spectra of DNA-dispersed single-walled carbon nanotubes (SWCNT). (**a**) VIS-NIR absorbance spectra of (GT)_10_-dispersed SWCNT. (**b**) NIR-fluorescence emission spectra of (GT)_10_-dispersed SWCNT after the addition of 0 M (black) and 100 nM dopamine (red). (**c**) NIR-fluorescence emission spectra of (GT)_10_-dispersed SWCNT after the addition of 0 M (black) and 100 nM epinephrine (red). (**d**) NIR-fluorescence emission spectra of (GT)_10_-dispersed SWCNT after the addition of 0 M (black) and 100 nM norepinephrine (red).

(6,5)-chirality enriched SWCNTs were dispersed using tip-ultrasonication with ten different oligonucleotides in 1× PBS (pH 7.4). The corresponding absorbance and NIR-fluorescence emission spectra are shown for (GT)_10_ as an example in Figure 2.

**Figure A1:**
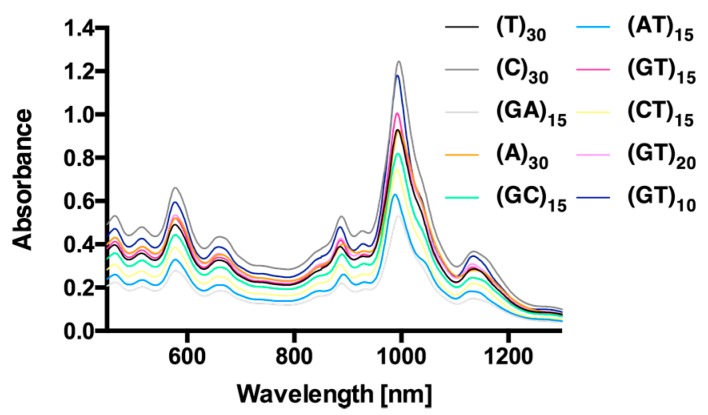
VIS-NIR absorbance spectra of different DNA-dispersed SWCNT samples. VIS-NIR absorbance spectra of DNA-oligonucleotide-dispersed single-walled carbon nanotubes (SWCNT) after centrifugation (see the Materials section) and before concentration adjustment. The (6,5)-peak at around 991 nm was used for concentration estimation and adjustment.

The absorbance spectra (Figure 2a and Figure A1) clearly show that it is possible to disperse the otherwise insoluble carbon nanotubes with all tested DNA sequences. For the fluorescence measurement with an excitation wavelength of 560 nm, SWCNT-DNA conjugates were adjusted in concentration to 0.1 nM according to their absorbance at the S_11_ peak at around 991 nm [31].

Figure 2b–d shows the representative fluorescence spectra of (GT)_10_-dispersed SWCNT before and after the addition of 100 nM dopamine, epinephrine, and norepinephrine. Between the three neurotransmitters, which were selected due to their structural similarity to dopamine, there is a clear difference in fluorescence intensity modulation at this concentration. Interestingly, most SWCNT-DNA conjugates responded to catecholamines by an increase of fluorescence. However, the absolute changes depend strongly on DNA sequence and structure of the analyte. This is in agreement with previous studies and indicates that these SWCNT-DNA conjugates can serve as sensors for catecholamine neurotransmitters [5,27]. However, the limits of detection and the dynamic range remained unclear, which is important if there are multiple catecholamines present in a sample.

### 3.2. Dose-Response Curves for Dopamine, Epinephrine, and Norepinephrine

**Figure 3:**
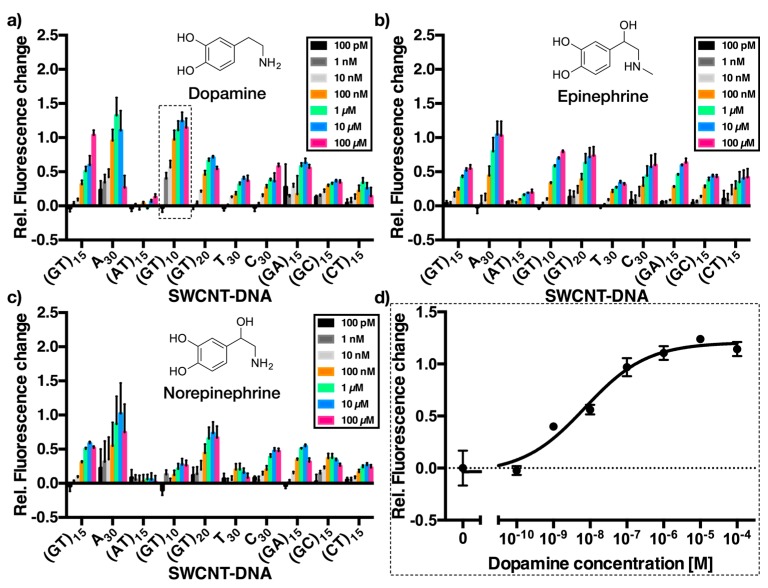
Dose-response curves of different catecholamine SWCNT/DNA sensors. Relative fluorescence change of the sensors upon addition of increasing concentrations (0, 100 pM, 1 nM, 10 nM, 100 nM, 1 μM, 10 μM, 100 μM) of dopamine (**a**), epinephrine (**b**), and norepinephrine (**c**). The *x*-axis shows different DNA sequences used to functionalize the SWCNTs. Here, the (6,5)-SWCNTs responses were evaluated. Errors are standard deviations (*n* = 3). (**d**) An example dose-response plot for dopamine recognition of SWCNT-(GT)_10_. Errors are standard deviations (*n* = 3).

A good sensor should not only be sensitive for its respective analyte, but also show high selectivity for its target and exclude biologically-relevant interfering molecules. To evaluate, whether the sensors for dopamine built from SWCNTs and oligomeric DNA sequences are suitable for the desired kind of discrimination between these neurotransmitters, we took the aforementioned pool of sensors and collected dose-response curves for each of them against dopamine, epinephrine, and norepinephrine. The resulting relative changes in fluorescence for each combination are depicted in Figure 3.

The data shown in Figure 3 contains all the information to evaluate sensor performance. The first and most obvious observation is that almost all SWCNT-DNA sensors react upon exposure to dopamine, epinephrine, and norepinephrine with an increase in NIR fluorescence. A_30_, (GT)_10_ and (GT)_20_ show the largest relative fluorescence increase for dopamine and epinephrine, while (GT)_10_-dispersed SWCNT only react minimally to norepinephrine. Interestingly, SWCNT-(AT)_15_ does not show any change in fluorescence in case of dopamine and norepinephrine and only a small change to epinephrine. However, for most analytical tasks it is important to consider relevant concentrations. For example, the maximum concentration of 100 μM used in these assays is high and will most likely not be reached (for long periods of time) in biological scenarios [34]. All sensors respond to catecholamines with different magnitudes and they saturate at different concentrations. Therefore, it should be possible to discriminate between different neurotransmitters at concentrations << 100 μM (see Figure 3).

**Figure 4:**
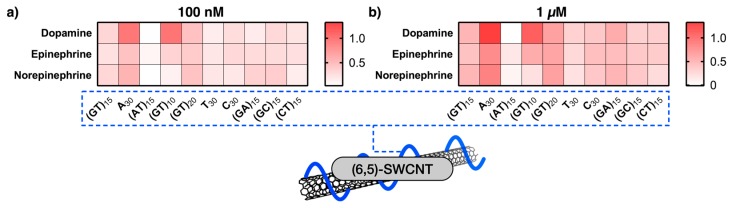
Sensitivity versus selectivity at low catecholamine concentrations. Relative fluorescence change of sensors upon addition of 100 nM (**a**) and 1 μM (**b**) of dopamine, epinephrine, and norepinephrine in the range from 0 (i.e., no change in fluorescence intensity) to 1.3 (equaling 130% fluorescence increase). Here, the (6,5)-SWCNTs responses at 987 nm were evaluated.

The heatmap in Figure 4 illustrates the relative NIR-fluorescence change of DNA-dispersed (6,5)-SWCNTs upon exposure to 100 nM and 1 μM dopamine, epinephrine and norepinephrine. Especially A_30_, (GT)_10_ and (AT)_15_ exhibit a different response to the three different neurotransmitters at these concentrations. This presentation of the response data is different from Figure 3 because it sets the focus on smaller concentrations, which are most likely more relevant in many analytical scenarios. For example, the static concentration of catecholamine neurotransmitters in brain tissue is in the order of 1–50 nM [35]. In contrast, concentrations of dopamine in vesicles reach values >100 mM [11]. When they are released, dopamine levels are very high for the first few milliseconds but decline very quickly to the nM regime [30]. Therefore, the effective mean concentration that is seen by a sensor during acquisition is typically in the range depicted in Figure 4 (1 nM–1 μM). Consequently, response data in this concentration regime provide a more realistic picture than at higher concentrations, at which most sensors already saturate. The results shown in Figure 4 indicate that for example at catecholamine concentrations of 100 nM the SWCNT-(GT)_10_ sensor is able to distinguish between dopamine and epinephrine/norepinephrine as the corresponding relative fluorescence intensity changes are 0.97 (±0.14) vs. 0.33 (±0.02)/0.12 (±0.07).

**Figure A2:**
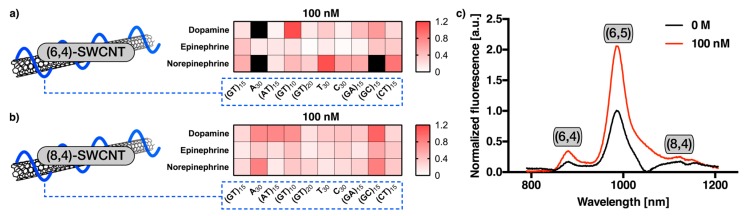
Chirality-dependence of the relative fluorescence change upon exposure to neurotransmitters. Relative fluorescence change of the sensors upon addition of 100 nM of dopamine, epinephrine and norepinephrine in the range from 0, i.e., no change in fluorescence intensity, to 1.2 equaling 120% fluorescence increase. Here, (6,4)-SWCNTs responses at 885 nm (**a**) and (8,4)-SWCNTs responses at 1122 nm (**b**) were evaluated. Black color indicates off-scale fluorescence changes presumably due to the small signal to noise ratios for the (6,4)-SWCNTs. (**c**) Representative fluorescence spectrum (SWCNT-(GT)_10_ and dopamine) and assignment of the nanotube chiralities.

As already shown by Salem et al., the chirality of the SWCNTs has an influence on SWCNT/DNA sensors similar to those presented in this work [36]. This behavior can be expected because different chiralities/diameters should cause different macromolecule adsorption (organic phases) and, therefore, different sensor responses. The corresponding heatmaps for the (6,4)- and (8,4)-SWCNT species are provided in Figure A2. Both chiralities also show increasing fluorescence at ≈885 nm and ≈1122 nm, respectively. The relative fluorescence increase, however, differs quite significantly (Figure A2). We attribute these large differences to a chirality dependence but the spectra consist of at least three nanotube species (6,4), (6,5), and (8,4) and the absolute differences might, therefore, be convoluted. Additionally, the experiments were performed for constant (6,5)-SWCNT concentrations (0.1 nM), adjusted via the (6,5)-S_11_ peak. The SWCNT concentration can have an influence on the absolute fluorescence values/changes and different dispersion yields by different DNA-oligonucleotides could affect it [5]. In the future, our studies could be extended to highly-purified (6,4) and (8,4) SWCNT species to study chirality dependence in greater detail.

### 3.3. Dissociation Constants and Limits of Detection for Different Neurotransmitter-Sensors

The recognition of catecholamines by SWCNT-DNA sensors can be seen as a bimolecular reaction:

(1) S+A⇌SA.

Here, the sensor *S* reacts with an analyte *A* to form a sensor/analyte complex *SA*. To get an idea of the dynamic range and the limits of detection for the different sensor-analyte combinations, the dose-response curves (Figure 3) were fitted using Equation (2):

(2) $$Y=y_{min}+\frac{y_{max}-y_{min}}{1+10^{\left(\log\left(K_{D}\right)-X\right)\times HillSlope}}$$

**Figure A3:**
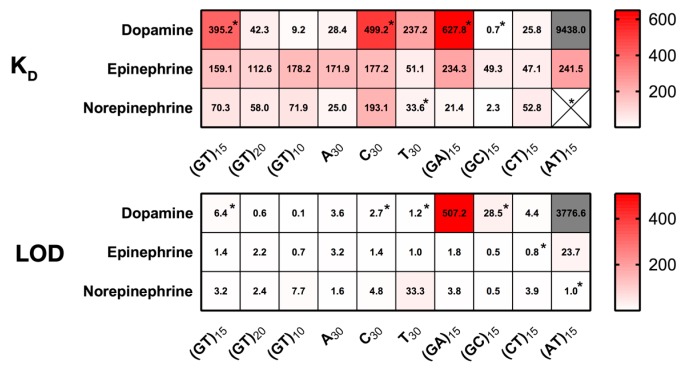
Overview of *K*_d_ and LOD values obtained from the dose-response curves for dopamine, epinephrine, and norepinephrine. * No clear (sigmoidal) fit possible.

The obtained dissociation constants from this logistic fit are shown in Table 1 and in a heatmap (Figure A3). In addition, Table 1 also contains the limits of detection extracted from each dose-response curve for every sensor-analyte combination.

The results show that there are large differences even though all sensors respond to catecholamines. The *K*_d_-values vary from 2.3 nM to 9.4 μM and the limits of detection from 0.5 to 507.2 nM. This span of six (four) orders of magnitude demonstrates the large influence of oligonucleotide sequence. *K*_d_ values give an idea of where the middle of the dynamic range of the sensor is and consequently for which analytical task it can be used.

Certain *K*_d_ values obtained for dopamine have larger confidence intervals due to poor fitting/non-sigmoidal dose-response curves. Nevertheless, the dissociation constants for epinephrine in general exceed those of dopamine and norepinephrine. In detail, especially SWCNT-(GT)_10_, SWCNT-(A)_30_, and SWCNT-(AT)_15_ seem to be well suited for discrimination between the three catecholamines, which are structurally very similar as they share the same catechol-moiety. By using MD simulations it was previously shown that the two hydroxy groups of the catechol-moiety interact with phosphate groups of the DNA backbone on SWCNT/DNA sensors [5]. Our results show fluorescence increases for all three neurotransmitters but the magnitude and the onset of saturation varies by orders of magnitude. This observation might be explained by differences between those three neurotransmitters (e.g., primary vs. secondary amine, size, and charge).

### 3.4. Detection of Dopamine with Single Carbon Nanotube Sensors in the Presence of Homologues

**Figure 5:**
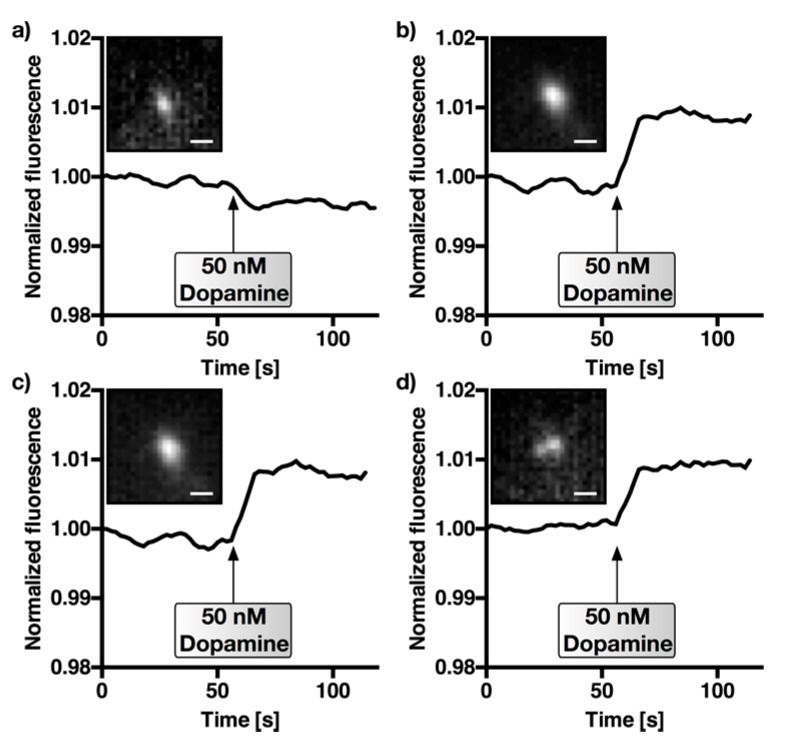
Detection of dopamine in the presence of norepinephrine. NIR fluorescence images of single (SWCNT)-DNA sensors. Here, 50 nM dopamine was added (*t* = 60 s) in the presence of 50 nM norepinephrine in phosphate-buffered saline. (**a**) Control experiment using SWCNT-(GA)_15_, which showed a lower affinity/selectivity for dopamine (see Figure 3 and Figure 4). (**b**–**d**) Three SWCNT-(GT)_10_-traces showing a change in fluorescence intensity upon dopamine addition. Insets show the fluorescence images of the single sensors at *t* = 0. Fluorescence counts were normalized. Scale bar = 1 μm.

One very important aspect of a sensor is the need to detect the analyte of interest (e.g., dopamine) in the presence of other relevant analytes, which can be very similar in structure. After having determined the dissociation constants and limits of detection for different SWCNT-DNA sensors for dopamine, epinephrine, and norepinephrine, the next step was to verify it in a realistic scenario with nM concentrations of dopamine and a homologue. SWCNT-(GT)_10_ sensors were immobilized on a glass surface by physisorption and the NIR response of single sensors to dopamine (50 nM) in a background of norepinephrine (50 nM) was quantified. SWCNT-(GT)_10_ sensors were used because the *K*_d_-values and responses (Figure 3 and Figure 4.) indicated that these sensors respond to dopamine, but not to norepinephrine, at this concentration. The results including the control using the lower-affinity sensor (GA)_15_ as a control are shown in Figure 5.

**Figure A4:**
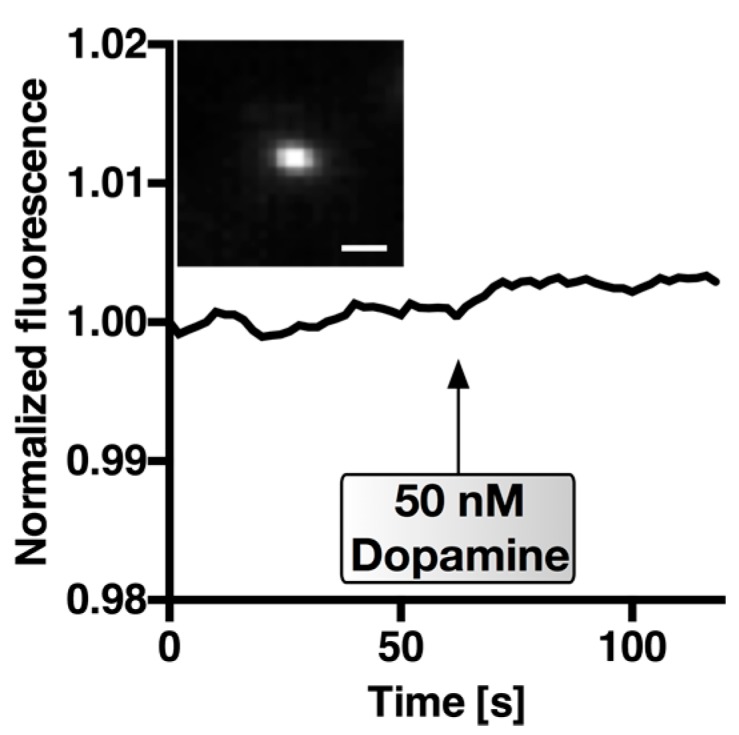
Control experiment for the detection of dopamine in the presence of norepinephrine. NIR fluorescence images of single (SWCNT)-DNA sensors. Here, 50 nM dopamine was added (*t* = 60 s) in the presence of 50 nM norepinephrine in phosphate-buffered saline. Control experiment using SWCNT-(AT)_15_, which showed a lower affinity/selectivity for dopamine (see Figure 3 and Figure 4). Insets show the fluorescence image of the single sensor at *t* = 0. Fluorescence counts were normalized. Scale bar = 1 μm.

Single SWCNT-(GT)_10_ sensor particles responded to dopamine in the presence of norepinephrine, which indicates that it is possible to discriminate those neurotransmitters, at least at 50 nM concentrations. In contrast, it was not possible to monitor the addition of dopamine after 60 s using neither the control sensor SWCNT-(GA)_15_ nor SWCNT-(AT)_15_ (see Figure A4) for which the results of Figure 3 and Figure 4 suggested a lower selectivity/affinity. The overall change in fluorescence intensity was not as high as in the solution-based experiments (Figure 3), which can be attributed to a different level of background and an impact of the surface on sensor responses. These NIR fluorescence microscopy experiments provide a spatial resolution and are closer to applications of these sensors for imaging of neurotransmitters in cell networks.

**Figure 6:**
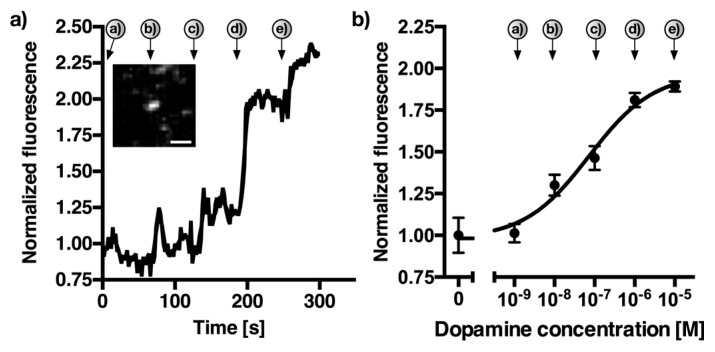
Dynamic detection of dopamine in the presence of norepinephrine. NIR fluorescence image of a single (SWCNT)-DNA sensor. Here, 1 nM to 10 μM dopamine was added approx. each 60 s (a = 1 nM, b = 10 nM, c = 100 nM, d = 1 μM, e = 10 μM) in the presence of 50 nM norepinephrine in phosphate-buffered saline. (**a**) Example SWCNT-(GT)_10_-trace showing a change in fluorescence intensity upon dopamine addition. Inset shows the NIR-fluorescence image of the single sensor at *t* = 300 s. Fluorescence counts were normalized. Scale bar = 1 μm. (**b**) Dose-response curve generated from the mean fluorescence changes of eleven different SWCNT-(GT)_10_ sensors between each addition-point of dopamine. Errors are standard deviations (*n* = 11).

To further evaluate the detection of dopamine with a 50 nM background of norepinephrine, another experiment similar to Figure 5 was carried out. However, in this case we gradually increased the dopamine concentration from 1 nM to 10 μM while maintaining a constant 50 nM norepinephrine background. Figure 6 shows the increasing fluorescence for single SWCNT-(GT)_10_ sensors, as well as the calibration curve generated from *n* = 11 sensors. The arrows denote the addition of dopamine (a = 1 nM, b = 10 nM, c = 100 nM, d = 1 μM, e = 10 μM).

The effective *K*_d_ increased to 72.9 nM compared to the solution-based value of 9.2 nM without the presence of 50 nM norepinephrine, while the limit of detection under these conditions changed from 0.1 nM to 4.9 nM. These results are very promising as they indicate that dopamine can be detected in the presence of realistic concentrations of the homologous neurotransmitter norepinephrine.

## 4. Discussion

The properties of nanomaterials depend strongly on their immediate chemical environment. In many cases this is an organic phase (corona), such as the DNA-oligonucleotides used in this work. This organic phase is not only important for dispersion of the hydrophobic SWCNTs, but also for interactions and recognition of other molecules. For sensors this is obviously clear and it is of general relevance when nanoparticles interact with their environment for example during nanoparticle growth or when studying or tailoring cell-nanoparticle interactions [37,38,39,40,41].

In this study we used ten different DNA-oligonucleotides to non-covalently modify (6,5)-enriched SWCNTs. These SWCNT-DNA conjugates were evaluated in terms of recognition ability of the three catecholamine neurotransmitters dopamine, epinephrine, and norepinephrine. They are important neurotransmitters, but so far it is extremely difficult to distinguish them when they are released by cells. As shown in Figure 3, almost all of the generated SWCNT-DNA conjugates showed increasing NIR fluorescence with increasing catecholamine concentration (range between 100 pM and 100 μM). One exception at this point is SWCNT-(AT)_15_, which did not show any response to dopamine or norepinephrine, and only a small increase to epinephrine. This behavior could possibly arise due to the self-complementarity, which leads to DNA-duplex formation in aqueous solution and on the SWCNT surface lowering its flexibility and thus hindering analyte-recognition. Another result are the special sensing capabilities of A_30_- and (GT)_10_-SWCNTs. Both of them exhibited a large relative fluorescence intensity change upon exposure to dopamine in comparison to both epinephrine and norepinephrine. In addition, as shown in Figure 4, they responded at much lower concentrations of dopamine than that of (nor)epinephrine, which can also be deduced from the measured *K*_d_ values summarized in Table 1. These two sequences might be very useful to discriminate between these chemically very similar analytes. The NIR fluorescence microscopy experiments of the (GT)_10_-SWCNT sensor showed, for single immobilized sensors, that low nM concentrations of dopamine are detected in the presence of equimolar amounts of norepinephrine. This result shows how the evaluation of dose-response curves (*K*_d_ and LOD values) helps to identify selective sensors and tune them for a specific application. In the future these insights may also be used in ratiometric sensing approaches with single-chirality SWCNT samples that emit light at different wavelengths.

The ‘library’ of ten DNA sequences led to sensors with *K*_d_ differences of up to six orders of magnitude. This is surprising because there was no rationale behind choosing those ten specific sequences. It is very likely that larger libraries and a screening or high-throughput approach could identify even more selective and sensitive sensors.

## 5. Conclusions

This study reveals the dose-response curves of several SWCNT/DNA sensors for the neurotransmitters dopamine, epinephrine, and norepinephrine. We find large differences in terms of dissociation constants and limits of detection for different DNA sequences and identify a sensor that can discriminate between the structural homologues dopamine and norepinephrine. These sensors can be used for biological studies that aim to distinguish these chemically very similar neurotransmitters. Our results also show that varying the organic phase around carbon nanotubes is a versatile approach to identify new sensors and to select the most selective and sensitive ones for specific analytical applications.

## Figures and Tables

**Table 1:** Overview of the different dissociation constants (*K*_d_) and limits of detection (LOD) values obtained for each SWCNT-DNA sensor-neurotransmitter combination. Both *K*_d_ and LOD are in nmol/L.

	NT	(GT)_15_	(GT)_20_	(GT)_10_	A_30_	C_30_	T_30_	(GA)_15_	(GC)_15_	(CT)_15_	(AT)_15_
*K*_d_	D	395.2 *	42.3	9.2	28.4	499.2 *	237.2	627.8 *	0.7 *	25.8	9438
	E	159.1	112.6	178.2	171.9	177.2	51.1	234.3	49.3	47.1	241.5
	N	70.3	58	71.9	25	193.1	33.6 *	21.4	2.3	52.8	-*
LOD	D	6.4 *	0.6	0.1	3.6	2.7 *	1.2 *	507.2	28.5 *	4.4	3776.6
	E	1.4	2.2	0.7	3.2	1.4	1.0	1.8	0.5	0.8 *	23.7
	N	3.2	2.4	7.7	1.6	4.8	33.3	3.8	0.5	3.9	*

* No clear (sigmoidal) fit possible. *K*_d_: Dissociation constant. LOD: Limit of detection definition used = 3× standard error at c = 0 nM. D: Dopamine. E: Epinephrine. N: Norepinephrine.
